# Supplementary figures and images for: Weekly Oral Tenofovir Alafenamide Protects Macaques from Vaginal and Rectal Simian HIV Infection
Source: Pharmaceutics. 2024 Mar 11;16(3):384. doi: 10.3390/pharmaceutics16030384 (PMC10974356; doi:10.3390/pharmaceutics16030384)

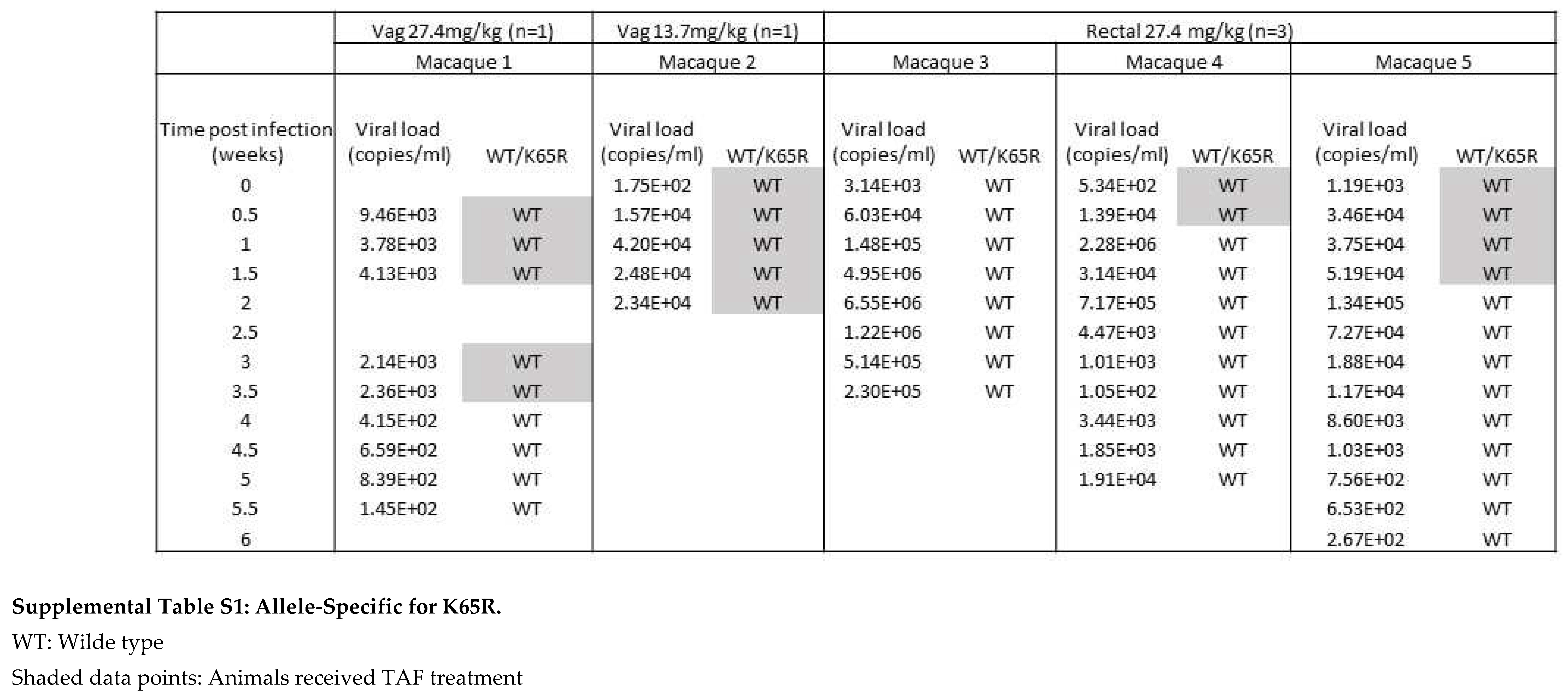

Supplement: Supplementary file 1 [file pharmaceutics-16-00384-s001.zip › pharmaceutics-2840901-supplementary.jpg]
